# Usability Testing of a Digital Assessment Routing Tool for Musculoskeletal Disorders: Iterative, Convergent Mixed Methods Study

**DOI:** 10.2196/38352

**Published:** 2022-08-30

**Authors:** Cabella Lowe, Mitchell Browne, William Marsh, Dylan Morrissey

**Affiliations:** 1 Centre for Sports & Exercise Medicine William Harvey Research Institute Queen Mary University of London London United Kingdom; 2 Risk and Information Systems Research Group School of Electronic Engineering and Computer Science Queen Mary University of London London United Kingdom; 3 Department of Physiotherapy Barts Health NHS Trust London United Kingdom

**Keywords:** mobile health, mHealth, eHealth, digital health, digital technology, musculoskeletal, triage, physiotherapy triage, usability, acceptability, mobile phone

## Abstract

**Background:**

Musculoskeletal disorders negatively affect millions of patients worldwide, placing significant demand on health care systems. Digital technologies that improve clinical outcomes and efficiency across the care pathway are development priorities. We developed the musculoskeletal Digital Assessment Routing Tool (DART) to enable self-assessment and immediate direction to the right care.

**Objective:**

We aimed to assess and resolve all serious DART usability issues to create a positive user experience and enhance system adoption before conducting randomized controlled trials for the integration of DART into musculoskeletal management pathways.

**Methods:**

An iterative, convergent mixed methods design was used, with 22 adult participants assessing 50 different clinical presentations over 5 testing rounds across 4 DART iterations. Participants were recruited using purposive sampling, with quotas for age, habitual internet use, and English-language ability. Quantitative data collection was defined by the constructs within the International Organization for Standardization 9241-210-2019 standard, with user satisfaction measured by the System Usability Scale. Study end points were resolution of all grade 1 and 2 usability problems and a mean System Usability Scale score of ≥80 across a minimum of 3 user group sessions.

**Results:**

All participants (mean age 48.6, SD 15.2; range 20-77 years) completed the study. Every assessment resulted in a recommendation with no DART system errors and a mean completion time of 5.2 (SD 4.44, range 1-18) minutes. Usability problems were reduced from 12 to 0, with trust and intention to act improving during the study. The relationship between eHealth literacy and age, as explored with a scatter plot and calculation of the Pearson correlation coefficient, was performed for all participants (*r*=–0.2; 20/22, 91%) and repeated with a potential outlier removed (*r*=–0.23), with no meaningful relationships observed or found for either. The mean satisfaction for daily internet users was highest (19/22, 86%; mean 86.5, SD 4.48; 90% confidence level [CL] 1.78 or –1.78), with nonnative English speakers (6/22, 27%; mean 78.1, SD 4.60; 90% CL 3.79 or –3.79) and infrequent internet users scoring the lowest (3/22, 14%; mean 70.8, SD 5.44; 90% CL 9.17 or –9.17), although the CIs overlap. The mean score across all groups was 84.3 (SD 4.67), corresponding to an *excellent* system, with qualitative data from all participants confirming that DART was simple to use.

**Conclusions:**

All serious DART usability issues were resolved, and a good level of satisfaction, trust, and willingness to act on the DART recommendation was demonstrated, thus allowing progression to randomized controlled trials that assess safety and effectiveness against usual care comparators. The iterative, convergent mixed methods design proved highly effective in fully evaluating DART from a user perspective and could provide a blueprint for other researchers of mobile health systems.

**International Registered Report Identifier (IRRID):**

RR2-10.2196/27205

## Introduction

### Background

Musculoskeletal disorders (MSDs) are prevalent across all ages, have shown an increase in the global disease burden over the past decade [1-3], and are associated with increased life expectancy and reduced activity [4,5]. MSDs are leading contributors to years lived with disability, early work retirement, and reduced ability to participate socially [5]. In many countries, they present the most significant proportional reason for lost productivity in the workplace, leading to significant impacts on the Gross Domestic Product and health care costs [6,7].

In the United Kingdom, the MSD burden of care poses a significant financial challenge to the National Health Service (NHS), costing £4.76 billion (US $3.84 billion) of resources and using up to 30% of primary care physician visits annually [8,9]. A freedom of information request has revealed that the average waiting times for NHS musculoskeletal outpatient physiotherapy services exceeded 6 weeks in the year to April 2019, with some patients waiting 4 months for routine physiotherapy appointments [10]. Longer waiting times can result in delays to physiotherapy services, with detrimental effects on pain, disability, and quality of life for waiting patients [11,12], highlighting the need for a targeted policy response [3,13].

Reducing inconsistency in clinical pathway delivery, including unwarranted secondary care consultations and investigations, forms part of the “Getting It Right First Time (GIRFT)” national program implemented within the UK NHS and has demonstrated cost reduction across the musculoskeletal pathway, particularly relevant in overburdened health care systems [14] Musculoskeletal triage as a single point of entry is effective in improving user satisfaction, diagnostic agreement, appropriateness of referrals, and reduction in patient waiting times [15], where it has been demonstrated to be effective using several methods by a range of clinicians [16-18]. However, using clinicians to provide MSD triage carries its own challenges [19].

Mobile health (mHealth), defined by the World Health Organization as a medical or public health practice that is supported by mobile devices [20], has seen rapid evolution and adoption, and currently, smartphone apps have the potential to make the treatment and prevention of diseases cost-efficient and widely accessible [21,22]. Optima Health has developed the mHealth Digital Assessment Routing Tool (DART) specifically for triaging MSDs, delivering a narrower but deeper assessment than that found with more generic symptom checkers. A digital alternative to clinician-led triage, which is able to replicate the same stratification of care and reduction in costs, is a desirable objective, although some mHealth tools have not demonstrated cost-effectiveness or have merely shown a shift in spending to another part of the health system [23]. It is also recognized that many mHealth apps fail to scale up from a prototype to successful implementation, with inattention to usability during the design and testing phases being identified as a potential cause of the high abandonment rate [24-27]. Although acknowledging usability is crucial in the design, development, testing, and implementation of mHealth apps [28-32], a consistent approach to testing has not yet been established, with researchers using a combination of different study methodologies [33].

An iterative, convergent mixed methods design was used to assess the usability of DART, using cyclical evaluation and improvement plus mixed methods to provide richness while quantifying use, maximizing usability, and therefore supporting system adoption [34]. The testing protocol for this study has been described in detail in a previous publication [35].

### DART Overview

**Figure 1:**
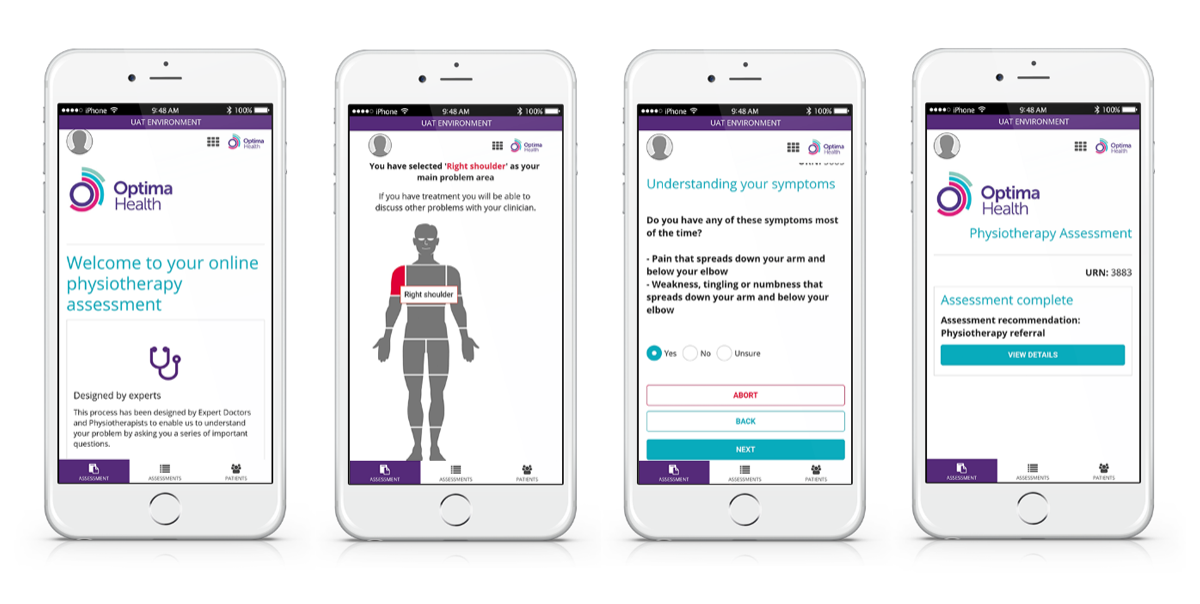
The Digital Assessment Routing Tool mobile health system.

DART is a first contact mHealth system comprising an algorithm distinguished by 9 body areas, providing the patient with a recommendation for the most appropriate level of intervention based on their responses (Figure 1). Screening for serious pathologies is completed at the start of the assessment, with less urgent medical referrals being identified as the patient passes through the questioning. The referrals recommended by the algorithm are configured to match the service provider’s local MSD pathways. DART typically signposts emergency or routine medical assessments, specific condition specialists, physiotherapy, self-management programs, and psychological support services.

**Figure 2:**
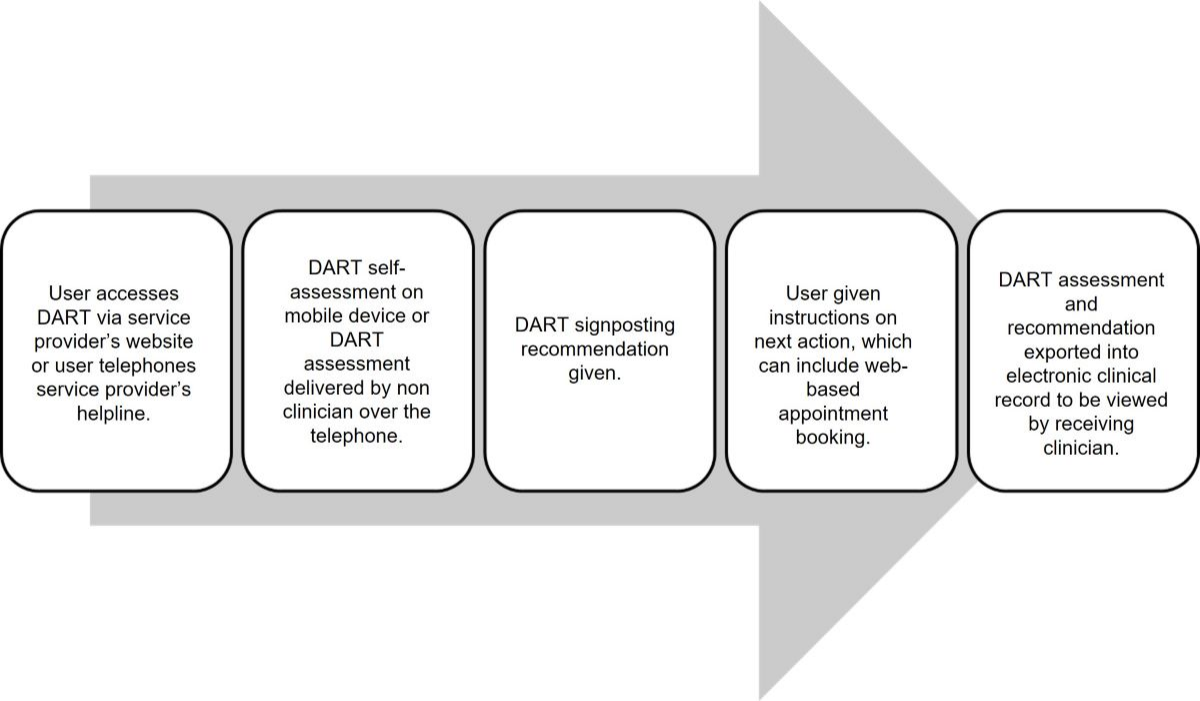
Integration of the DART mobile health system within an existing musculoskeletal disorder pathway. DART: Digital Assessment Routing Tool.

Integration of DART with the provider’s clinical record system means that assessment data and recommendations can be made instantly available to the receiving clinician. Using a link on the clinical provider’s website, DART can be accessed 24/7 using a mobile device or computer, directing users to care at an earlier stage of their injury than would be possible via a traditional clinician-led triage process (Figure 2). Alternatively, DART can be delivered over the telephone by a nonclinician. Reduction in treatment waiting times and reallocation of triage clinical resources to more complex assessments and management could hold significant benefits for the user and health care system.

### Previous Work

This usability study is part of a larger project, bringing DART from concept to implementation through a series of clinical and academic research work packages. Clinical algorithm validity was assessed by a panel of clinical experts using vignettes incorporating common MSD presentations, as well as red flags and complex presentations, with the panel deeming the validity to be sufficient to allow DART to proceed to further research studies. The protocol devised for this usability study went through a series of iterations within an internal review process, comprising the research project team and DART system developers to arrive at the final version [35]. The objective of this study was to optimize usability before evaluating the safety and effectiveness of DART through a randomized controlled trial, the pilot protocol for which has been published [36].

## Methods

### Study Design

**Figure 3:**
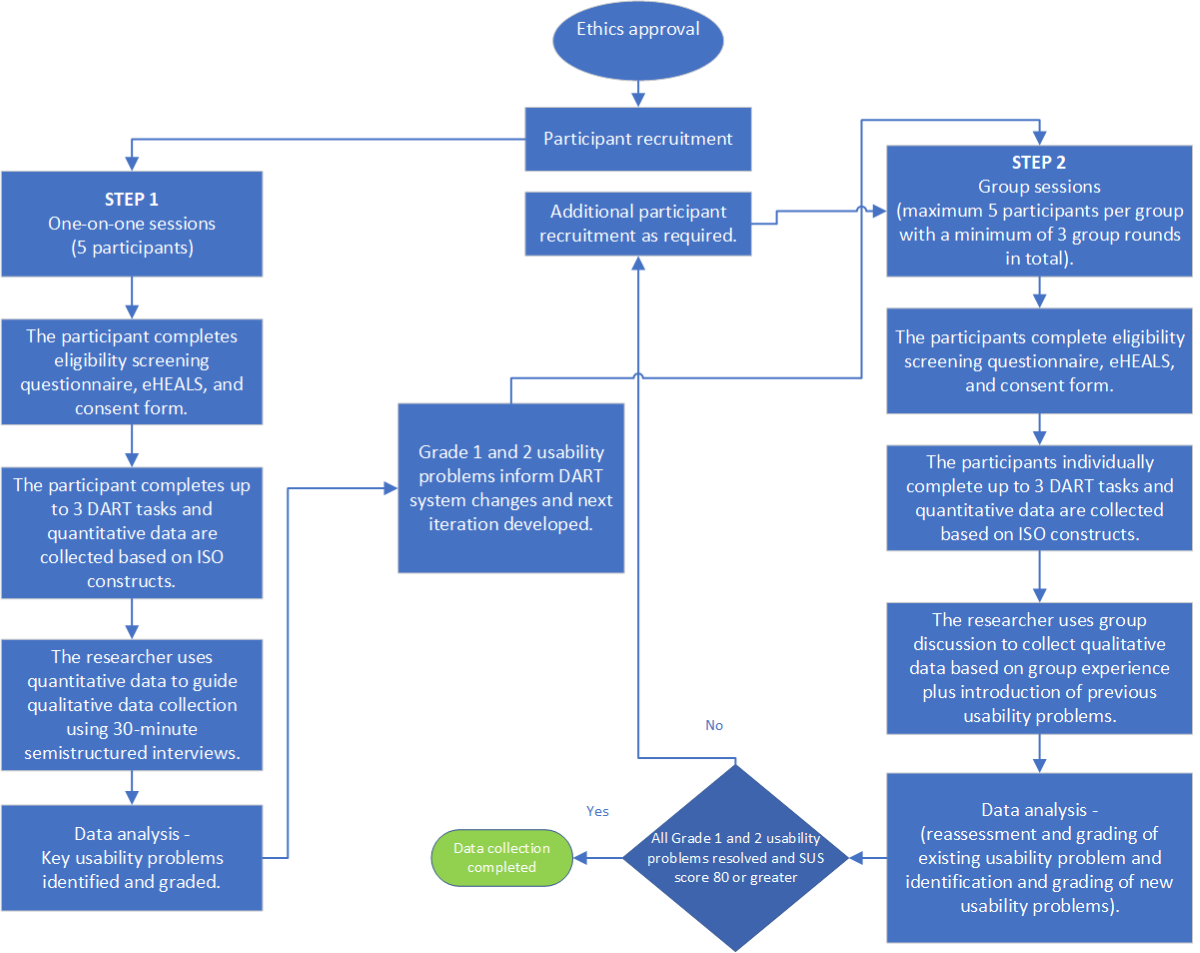
DART usability study iterative, convergent mixed methods design. New participants were recruited for each testing round. Participants raising specific issues in previous rounds were invited individually to review and provide feedback on changes. DART: Digital Assessment Routing Tool; eHEALS: eHealth Literacy Scale; ISO: International Organization for Standardization.

This study used an iterative, convergent mixed methods design, the protocol for which has been published elsewhere [35]. Step 1 involved in-depth interviews with 5 participants to identify key usability issues, followed by step 2, where group sessions captured greater diversity of data from a potential DART user population (Figure 3). Quantitative data collection was defined by the constructs of effectiveness, efficiency, and satisfaction within the International Organization for Standardization (ISO) 9241-210-2019 standard [30] and provided researchers with a focus for qualitative data collection during both steps. Accessibility was monitored throughout the testing process following the principles described in ISO 30071-1-2019 for embedding inclusion within the design process [31]. Mixed methods data collection and analysis continued cyclically through all rounds of testing until the fourth DART mHealth system iteration was found to perform according to the agreed criteria and the study end points of all grade 1 and 2 usability problems being resolved, as well as a mean System Usability Scale (SUS) score of ≥80, were achieved. The relationship between the likelihood to recommend a system and the mean SUS score has been found to be strongly correlated, and a score of ≥80 was chosen as a study end point as achievement of this threshold is considered to increase the probability of users recommending the system to a friend, therefore positively affecting adoption [37].

### Participant Recruitment

A stratified purposive sampling method was used to gather information from participants by using a sampling matrix and quotas [38], categorized by participant age, internet use, sex, and English for speakers of other languages (ESOL) groups—all of which are subgroups that have shown to contribute small differences in internet use [39]. For this study, “daily internet users” were defined as individuals who access the internet every day or almost every day, and “infrequent internet users” were those who were not daily users but had accessed the internet within the past 3 months [39]. Recruitment was conducted via flyers and emails to local community groups, Optima Health’s existing client base of employers and staff, and Queen Mary University of London students, as well as via social media. In the latter stages, snowballing yielded participants with characteristics of interest; study recruitment continued throughout the study process until the study end points were reached. Potential participants expressing an interest were sent a patient information sheet and consent form and had the opportunity to review this material before consenting to join the study. A total of 33 individuals expressed an interest in participating, of whom 22 (67%) enrolled in the study after meeting the screening criteria.

### Inclusion and Exclusion Criteria

The study participant inclusion criteria were as follows: (1) adults aged >18 years; (2) able to speak and read English; (3) living in the United Kingdom; (4) accessed the internet at least once every 3 months; (5) access to a smartphone, tablet, or laptop; and (6) current or previous experience of a musculoskeletal condition.

The study participant exclusion criteria were as follows: (1) significant visual or memory impairment sufficient to affect the ability to answer questions and recall information in an individual or group discussion setting; (2) medically trained musculoskeletal health care professional, such as a physician or physiotherapist; (3) relatives or friends of the researchers; and (4) Optima Health employees.

### Data Collection

Following the attainment of consent, participants completed a short questionnaire, including the eHealth Literacy Scale (eHEALS) [40], to provide demographic data and were given instructions by the researcher on how to log into the DART system test site. The first 5 participants in step 1 attended one-on-one video call interviews lasting up to 60 minutes where they could choose up to 3 existing or previous musculoskeletal conditions to complete assessments while being encouraged to give feedback using the concurrent think-aloud method [41]. Participant choice was not limited to specific body sites as usability features were synonymous across all 9 body sites. The participants in step 2 tested DART individually and then attended 30-minute video call group discussion sessions facilitated by the researcher.

**Figure 4:**
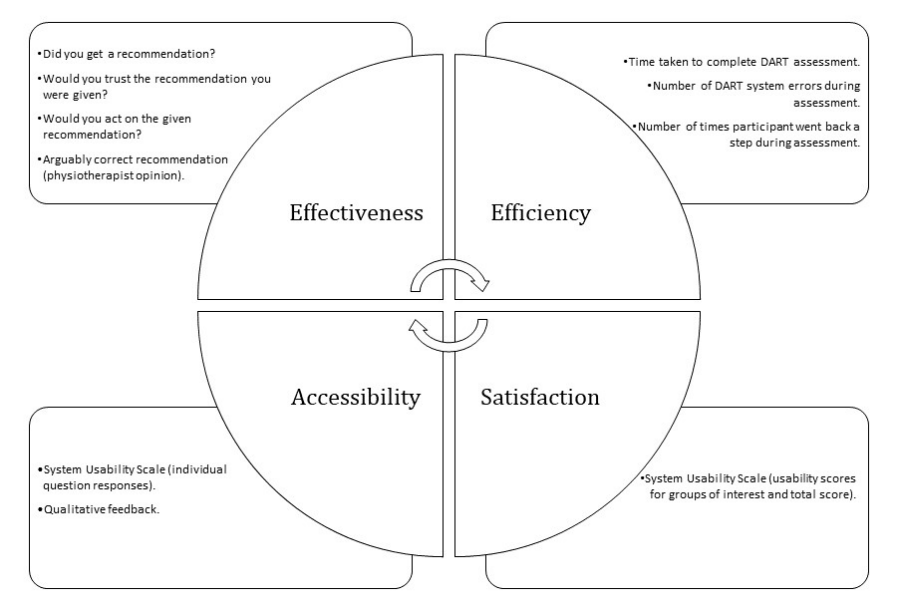
Data collection methods used to assess DART performance against the International Organization for Standardization 9241-210-2019 and International Organization for Standardization 30071-1-2019 standards constructs of effectiveness, efficiency, and satisfaction [30] and accessibility [31]. DART: Digital Assessment Routing Tool.

Assessing DART performance using satisfaction scales alone was not considered adequate; thus, data collection parameters were defined using the ISO constructs (Figure 4). Following their DART assessments, all participants completed a questionnaire and the SUS [42-44]. The researcher (physiotherapist with postgraduate MSD qualifications) assessed the clinical accuracy of the DART recommendation based on the diagnosis the participant had been given by their treating clinician. Quantitative data were also taken from the DART system itself.

Quantitative data aligned to the ISO 9241-210-2019 standard constructs were generated from participant questionnaires and DART system data, as shown in Figure 4. These informed the researcher’s qualitative data collection, aided by the use of a visual joint display, merging both types of data to illuminate not only usability problem themes but also potential system improvements (Multimedia Appendix 1). Qualitative data recorded during the interviews and group sessions were transcribed verbatim using the Otter transcription software (Otter.ai; automated video and audio transcription software) and checked for accuracy against the original recording. During group sessions, previous usability problems were introduced to participants to assess the impact of changes made to the previous iteration. In addition, users who raised specific issues in previous rounds were invited individually to review and feedback on changes. In addition to usability problems, any participant feedback on accessibility or positive aspects of DART was recorded.

**Figure 5:**
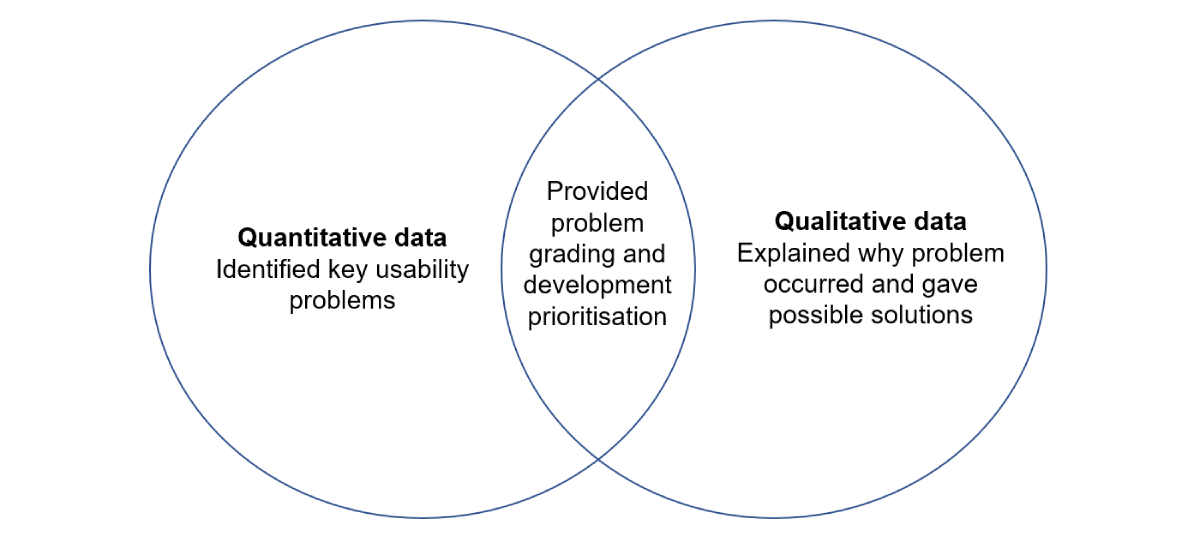
The convergent mixed methods design, where both data types are collected simultaneously to allow the analysis and grading of usability problems, thus informing the next system iteration.

Data analysis occurred after each round of testing and leveraged the strengths of the convergent mixed methods design to identify usability issues and inform the changes required for subsequent DART iterations (Figure 5). Of particular importance was the thematic analysis of qualitative data provided by real-world users, which ensured their views were included in the DART system development to improve usability.

Data analysis was performed to identify the overall satisfaction score and differences between groups (mean score, SD, and confidence level [CL]). Statistical analyses examined the relationship between participant age and eHealth literacy using Pearson correlations.

Restrictions imposed during the COVID-19 pandemic led to all data collection sessions being conducted remotely using Microsoft Teams videoconferencing software and web-based questionnaires.

### Ethics Approval

This study received approval from the Queen Mary University of London Ethics of Research Committee (QMREC2018/48/048) in June 2020.

### Data Analysis

Extending the convergent mixed methods design from data collection to analysis, the reporting used a weaving approach where usability problems were brought together on a theme-by-theme basis and presented through joint displays [45].

Quantitative data from web-based questionnaires and measures of efficiency from the DART system were analyzed and reported to identify key usability issues. Participant SUS raw scores were converted and analyzed by groups of specific interest (daily internet users, infrequent internet users, and ESOL), and the amalgamated mean score across all participants was converted into a percentile score to provide benchmarking against other web-based systems [46].

To minimize bias, quantitative data were collected by an independent researcher during the initial 5 semistructured interviews, and web-based questionnaires were used for the group sessions. Using a thematic analysis approach, qualitative data derived from transcripts of interviews and group sessions were reviewed and analyzed systematically by the 2 researchers independently. Patterns and clusters of meaning within the data were identified and labeled according to the area of system functionality. Data not directly related to the overall research question were excluded. The 2 researchers then worked together to agree and create a thematic framework with higher-order key usability themes able to address the research objective [47,48]. Data were indexed into usability problems of key importance to the study and quotes extracted for each subtheme, thus providing the details required to make the system changes needed to remove or mitigate grade 1 and 2 usability issues. The researchers, working independently initially and then together, arrived at a consensus and allocated a problem severity grade to each usability problem. This was obtained by considering the impact and frequency of the problem, leading to a decision on the risk of not addressing the problem versus the reward of correcting it [49] (Table 1). Once problems had been graded, matched system developments were passed to the DART system developers to guide the next iteration. Actions to address all grade 1 and 2 usability problems were completed for the next iteration, together with closely associated grade 3 and 4 problems if they fell within the scope of the development work. All usability problems remained on record and were reassessed after each round and, if necessary, regraded. Positive feedback about the system was also reported.

**Table 1:** Usability problem grading criteria, adapted from guidance issued by The Food and Drug Administration [49].

Grade	Impact	Frequency	Implications	Action
1	High	High, moderate, or low	Prevents effective use of the system	Address in next study iteration
2	Moderate or low	High or moderate	Affects the quality of system delivery	Address in next study iteration
3	Moderate or low	Low or moderate	Minor issues for several users or a small number of users highlighting concerns important to them	Document and address in later development
4	Low	Low	Small issues that, if resolved, could improve user satisfaction	Document and address in later development

### Statistical Analysis

The relationship between participant age and eHealth literacy was analyzed using Pearson correlations in Microsoft Excel (a spreadsheet with statistical analysis functionality) to identify user groups less likely to use DART successfully.

Differences in satisfaction scores were present between groups, with expert internet users having the highest mean score (mean 86.5, SD 4.48; 90% CL 1.78).

## Results

### Overview

A total of 22 participants were enrolled and completed the study (Table 2). The first testing round comprised 23% (5/22) of participants who completed qualitative “think-aloud” data collection led by a researcher familiar with the system and with training in the use of the method. It has been suggested that this relatively small number of participants is sufficient to expose 75% of usability issues, including all catastrophic problems, with further testing of subsequent iterations using new participants to identify less serious problems [50]. This proved to be the case, and data sufficiency was achieved. This was supported by a narrow study aim and the quality of dialog with the first 5 participants. The final sample size was not predefined and was re-evaluated after each round of results [51].

There was representation from all the groups of interest; however, not all quotas were met, and small sample sizes, especially infrequent internet users, resulted in a skew of data in favor of daily internet users. This compromised detailed statistical analyses across groups (Table 3).

**Table 2:** Participant characteristics (N=22).

Characteristic		Daily internet users	Infrequent internet users	ESOL^a,b^	All groups
Total sample, n (%)		19 (86)	3 (14)	6 (27)	22 (100)
**Age (years)**					
	Values, mean (SD)	47.6 (15.7)	55 (11.4)	41 (8.5)	48.6 (15.2)
	Values, range	20-77	47-68	31-55	20-77
Sex (male), n (%)		9 (41)	1 (5)	3 (14)	10 (46)
**eHEALS^c^ score**					
	Values, mean (SD)	29 (8)	25 (4)	26 (12.3)	28.8 (7.8)
	Values, range	8-38	21-29	8-37	(8-38)

^a^ESOL: English for speakers of other languages.

^b^All ESOL participants were also daily internet users.

^c^eHEALS: eHealth Literacy Scale.

**Table 3:** Recruitment matrix showing minimum quotas and number of participants recruited by characteristics of interest (N=22)^a^.

Characteristic		Daily internet user (n=19)		Infrequent internet user (n=3)	
		Quota	Enrolled, n (%)	Quota	Enrolled, n (%)
**Age (years)**					
	18-54	2-4	7 (37)	1-3	2 (67)
	55-74	2-4	10 (53)	1-3	1 (33)
	≥75	1-3	1 (5)	2-4	0 (0)
**Sex**					
	Male	Minimum 6	7 (37)	Minimum 4	1 (33)
	Female	Minimum 6	10 (53)	Minimum 4	2 (67)
**ESOL^b^**					
	Non-ESOL	Minimum 6	15 (79)	Minimum 6	3 (100)
	ESOL	Minimum 2	6 (32)	Minimum 2	0 (0)

^a^Total study participants quota was 20.

^b^ESOL: English for speakers of other languages.

We were interested to know whether the frequency of internet use, age, eHealth literacy, or being a speaker of English as a second language would affect DART usability, as these factors have been highlighted as potential variables in mHealth adoption [39]. There was a wide range of eHEALS scores across participants (mean 28.8, SD 7.8; 95% CI 25.1-32.3), with the highest score of 38/40 achieved by a daily internet user aged 27 years and the lowest score of 8/40 achieved by an ESOL daily internet user aged 31 years. The oldest participant (aged 77 years) achieved a score of 37/40, and the youngest participant (aged 20 years) scored 30/40.

**Figure 6:**
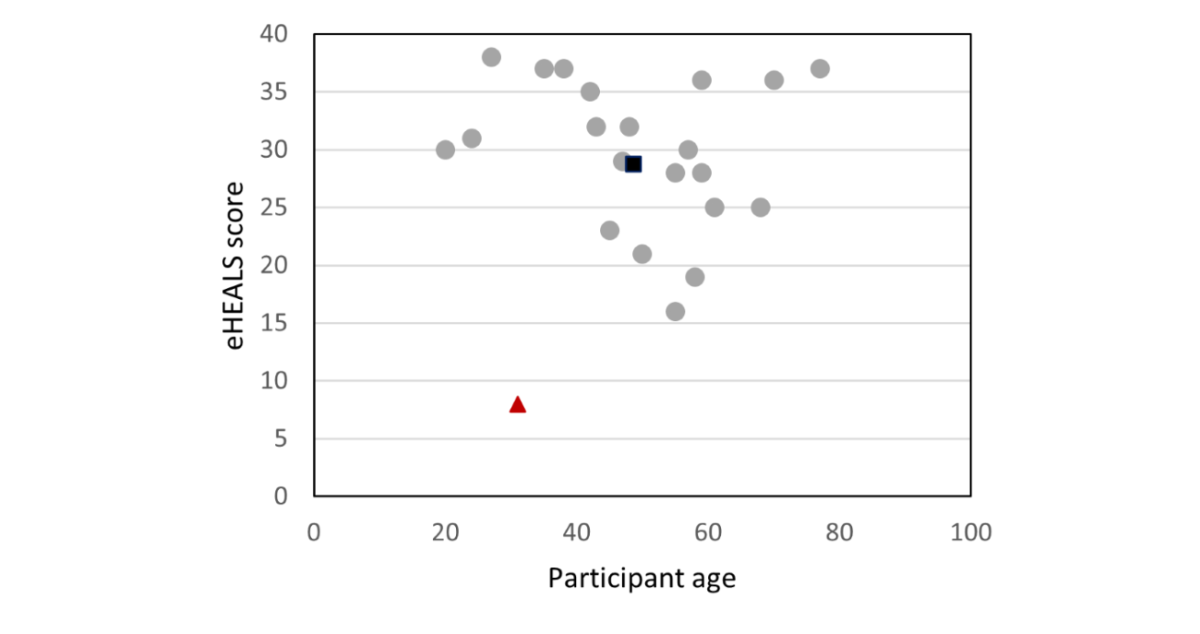
Relationship between age and eHealth literacy scores across all participants. (eHEALS scores range from 0 to 40 scale, with higher scores being better). eHEALS: eHealth Literacy Scale.

The relationship between eHealth literacy and age, as explored with a scatterplot and calculation of Pearson correlation coefficients, was performed for all participants (20/22, 91%; *r*=–0.2) and repeated with the potential outlier removed, as indicated in Figure 6 in red (19/22, 86%; *r*=–0.23), with no meaningful relationship observed or found for either.

**Figure 7:**
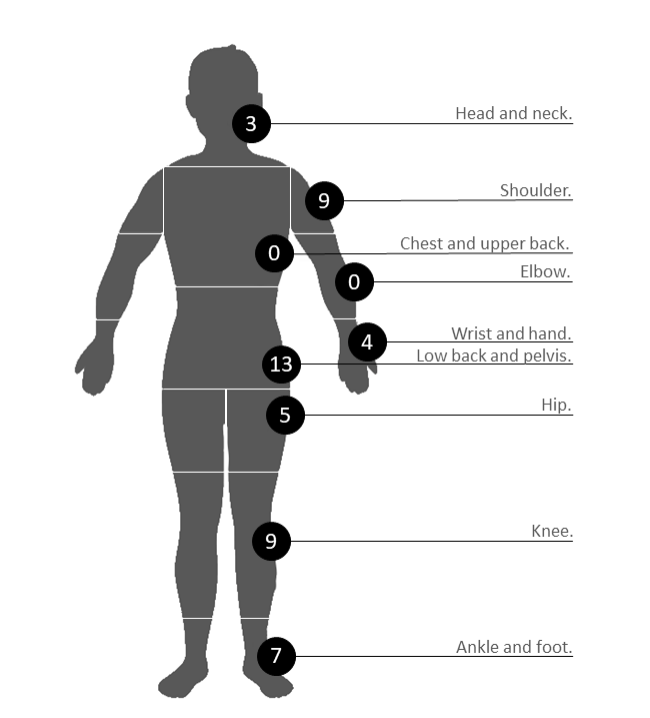
Body sites selected by participants. The number of selections represents the total of the front, back, and either side of a given body site. The Digital Assessment Routing Tool algorithms are designed to assess for musculoskeletal disorder conditions that occur or refer to pain in the selected body site.

A total of 50 assessments were completed by the 22 participants across a possible 9 body sites (Figure 7). The most frequently chosen body site was the low back and pelvis (13/22, 26%), followed by shoulder and knee (both 9/22, 18%). Two body sites were not selected by participants for testing: chest and upper back and elbow. Within a typical MSD triage service, these are often the least occurring body sites. However, the usability features are consistent with those of the other body regions; thus, it is unlikely that any new problems would have been identified through the selection of these pathways.

### Usability Problems

**Figure 8:**
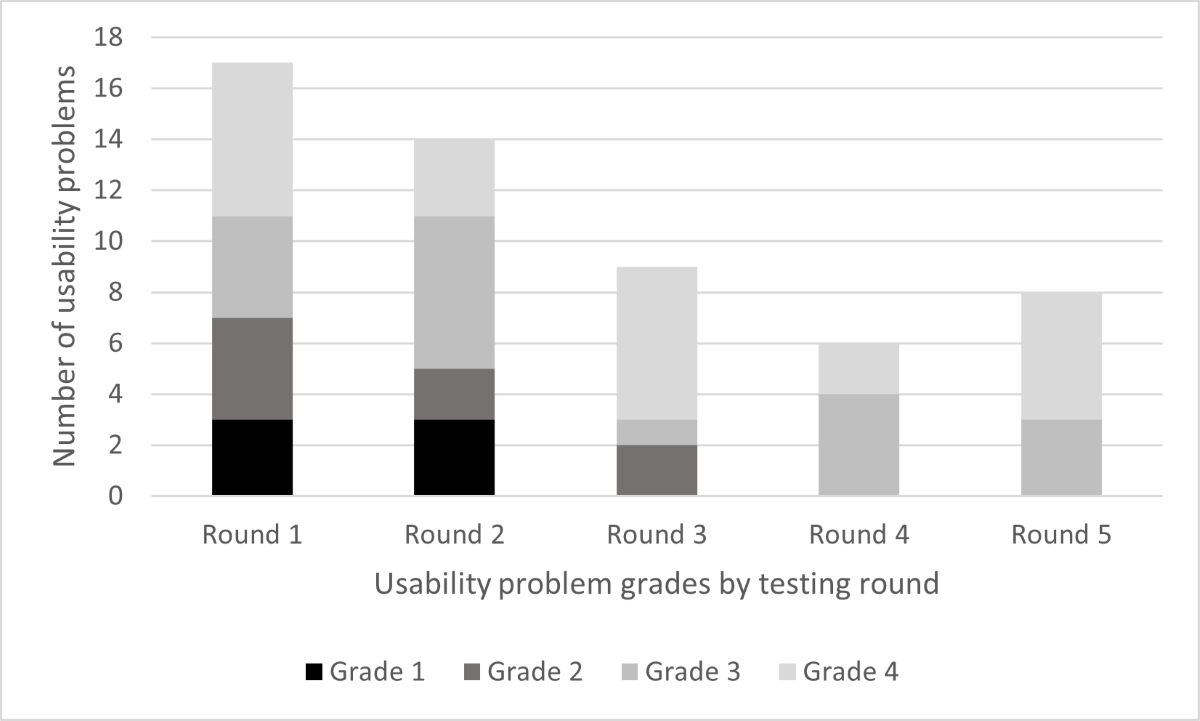
Number of usability problems across testing rounds by grade. The incidence and problem grading changed over the 5 rounds of testing, with grade 1 and 2 problems being negated or reduced to a lower grade. All grade 3 and 4 issues were documented, reviewed, and prioritized for future Digital Assessment Routing Tool development.

A total of 19 individual usability problems were identified across all 5 rounds of testing, of which 12 (63%) were initially classified as grade 1 or 2. These grades were either reduced or resolved over the iterations. DART iteration 4 was reviewed by participants during testing round 4, and no grade 1 or 2 usability problems were found. This was validated during testing round 5, and the study end points were achieved (Figure 8). 

**Figure 9:**
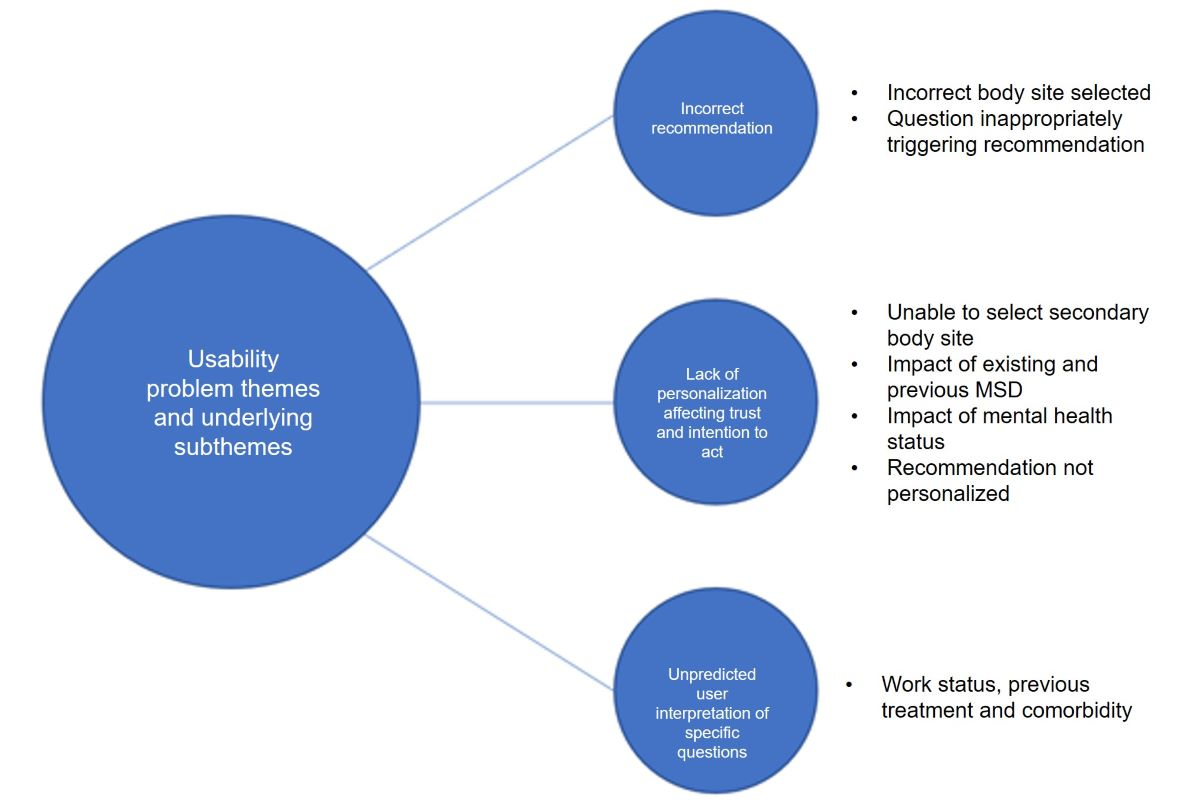
Grade 1 and 2 usability problem themes and underlying subthemes. MSD: musculoskeletal disorder.

Within the grade 1 and 2 usability problems, 3 main themes and 7 contributory subthemes were identified (Figure 9).

Over each of the 5 testing rounds, grade 1 and 2 usability problems were discussed with the participants and regraded (Table 4).

**Table 4:** Grade 1 and 2 usability problem themes, subthemes, and participant quotes over 5 rounds of testing^a^.

Underlying theme and subthemes			Usability problem grade of subtheme (testing round)		Participant quotes
**Theme 1: incorrect DART^b^ recommendation compared with expert opinion**					
	Incorrect body site selected	1 (1) 2 (2) 2 (3) 4 (4) 4 (5)		“If people are like me, they don't read things properly, especially at the beginning. Was there an option to start again, because people might mess up?” [DART005] “The only time that I felt slightly lacking in confidence was on the body site. That was the only time I wasn’t sure the system would grab the information I clicked.” [DART002]	
	Question inappropriately triggering recommendation—systemic inflammatory disease and central nervous system condition	1 (1) 1 (2) 0 (3) 0 (4) 0 (5)		“I think that came because I said that I'm stiff in the morning, that eight minutes or whatever.” [DART006] “So I read weakness, not severe weakness. So, I can stand on it and support my weight, but it just hurts like hell, rather than not being able to support myself.” [DART015]	
**Theme 2: lack of personalization affecting participants’ trust and intention to act**					
	Unable to select secondary body site	0 (1) 2 (2) 2 (3) 0 (4) 0 (5)		“I think if I could have put more evidence in, then I’d be more likely to follow the recommendation at the end because I think it was relevant to me.” [DART006] “I suppose you could differentiate slightly more between the source point of the pain and the consequences for your other limbs like, you know, I knew very well that it was bad at the back that was causing my inability to walk. So maybe distinction between primary pain and a secondary or referred pain might be useful.” [DART010]	
	Impact of existing and previous MSD^c^	2 (1) 0 (2) 0 (3) 0 (4) 4 (5)		“I don’t want to waste the GP’s time or my time waiting for an appointment to be told what I already know. So, in my two cases, it wasn’t so much about diagnosis is more of an okay, this has returned. We know the course of action.” [DART019] “I wasn't sure whether sometimes we’re talking about what it’s like when it’s really bad, or what it’s like in general.” [DART005]	
	Impact of mental health status	0 (1) 2 (2) 3 (3) 3 (4) 3 (5)		“I think one way of making it better is also seeing how it affects someone psychologically as well. I think that this is something which can sometimes be overlooked, but I think it's important to see how it is impacting on someone's emotional wellbeing?” [DART001] “When you seek sort of medical advice, or you have a condition that gives you worry and anxiety, probably you expect a little bit more than just sort of self-treatment.” [DART016]	
	Recommendations not sufficiently personalized	4 (1) 2 (2) 0 (3) 0 (4) 0 (5)		“I guess, it might need to be a little bit more personalized recommendations depending on what people choose.” [DART001] “I suppose the only thing that might dissuade people, would be that if they were users of it, and it came up with the same sort of end page every time.” [DART002]	
**Theme 3: participant difficulty in interpreting questions**					
	Specific questions (work status, previous treatment, and comorbidities)	2 (1) 3 (2) 4 (3) 0 (4) 4 (5)		“You were distinguishing between people who were employed, and people who are not employed. It just seemed to me as though there was quite a large category of people lumped together in that one box and maybe it would be better to differentiate them a bit more, so that they did actually tick retired or they ticked student” [DART010] “Where you were asked whether you'd had surgery or physio, it just was rather a broad question. I thought maybe it should have been a tick box for that to show which one you’d had.” [DART004] “You’re a little bit unsure about whether it’s really registered to your osteoporosis.” [DART010]	

^a^Usability problems were clustered into subthemes based on specific areas of DART functionality. Problem grades were reduced in severity over testing rounds as problems were negated or reduced during DART iterations (grade 1 is the most severe, and grade 4 is the least severe).

^b^DART: Digital Assessment Routing Tool.

^c^MSD: musculoskeletal disorder.

Construction of a joint display showed how different types of data were combined to assess performance against the ISO 9241-210-2019 constructs (Tables 5, 6, and 7).

**Table 5:** Display of qualitative data by International Organization for Standardization 9241-210-2019 standard constructs (effectiveness, efficiency, and satisfaction)^a^.

Construct and goal			Participant quotes
**Construct 1: effectiveness**			
	Assessment results for a recommendation being given	“I found it really user friendly and I found I could read the questions quite quickly and just give an answer and move on.” [DART018]	
	Assessment results for a correct clinical recommendation;	“I expected the area [selected body part] that I chose to change color, I would do it a different color, red or something like that.” [DART005]	
	Assessment of whether the participant would trust	“It might be easier if you just say have a secondary field to sort of like give your secondary issues as well. You know, sometimes it's just not, it's like the neck runs into the arm and lower parts, but it can be different things as well.” [DART014] “It might make people feel a bit more confident that they've done it right.” [DART015]	
	Assessment of whether the participant would act upon	“I think if I could have put more evidence in, then I’d be more likely to follow the recommendation at the end, because I think it was relevant to me.” [DART006]	
**Construct 2: efficiency**			
	Time taken to reach recommendation (minutes)	“It was very quick. And I quite like that it has one thing for one page, which is a very short question, it gives you a few options, and then you answer so you don't have to go through long text questions, one after the other. So, it just takes you very quickly step by step. And it's quite, I don’t know, for me, it was super easy and clear to answer questions.” [DART017]	
	DART^b^ system errors	“I found it really simple system to use very, very easy and had no problems at all.” [DART010B]	
	DART system backsteps	“That was a question about whether I’d been off work for a long time and if I’m employed or self-employed, something that I didn’t find quite straightforward.” [DART020]	
**Construct 3: satisfaction**			
	System Usability Scale score per round	“If I had this actual system, I would have saved £150 in cash and probably three months of pain had I been able to access it when I had my problems with my back.” [DART013] “It’s done me a favor actually, because I was in two minds whether to try and get a private injection, whether to go to an osteopath or physio. I think it might save me money in the long run.” [DART014]	

^a^Participant quotes provide a deeper understanding of system performance and usability problems.

^b^DART: Digital Assessment Routing Tool.

**Table 6:** Display of quantitative data by International Organization for Standardization 9241-210-2019 standard constructs (effectiveness, efficiency, and satisfaction) over 5 testing rounds^a^.

Construct, goal, and testing round					Result
**Construct 1: effectiveness^a^**					
	**Assessment results for a recommendation being given; participants in testing round achieving construct theme (%)**				
		Round 1		13 (100)	
		Round 2		11 (100)	
		Round 3		11 (100)	
		Round 4		10 (100)	
		Round 5		5 (100)	
	**Assessment results for a correct clinical recommendation; participants in testing round achieving construct theme (%)**				
		Round 1		11 (85)	
		Round 2		5 (45)	
		Round 3		11 (100)	
		Round 4		10 (100)	
		Round 5		10 (100)	
	**Assessment of whether the participant would trust; participants in testing round achieving construct theme (%)**				
		Round 1		13 (100)	
		Round 2		9 (82)	
		Round 3		11 (100)	
		Round 4		8 (80)	
		Round 5		5 (100)	
	**Assessment of whether the participant would act upon, n (%)**				
		Round 1		12 (92)	
		Round 2		8 (73)	
		Round 3		11 (100)	
		Round 4		8 (80)	
		Round 5		4 (80)	
**Construct 2: efficiency^b^**					
	**Time taken to reach recommendation (minutes)**				
		**Round 1**			
			Values, mean (SD)	Not recorded	
			Values, range	Not recorded	
		**Round 2**			
			Values, mean (SD)	5.7 (5.35)	
			Values, range	1-18	
		**Round 3**			
			Values, mean (SD)	5.4 (4.54)	
			Values, range	1-15	
		**Round 4**			
			Values, mean (SD)	3.5 (1.5)	
			Values, range	1-5	
		**Round 5**			
			Values, mean (SD)	7.4 (2.13)	
			Values, range	3-17	
		**All groups**			
			Values, mean (SD)	5.2 (4.44)	
			Values, range	1-18	
	**DART^c^ system errors**				
		Round 1		0	
		Round 2		0	
		Round 3		0	
		Round 4		0	
		Round 5		0	
	**DART system backsteps**				
		Round 1		1	
		Round 2		2	
		Round 3		2	
		Round 4		2	
		Round 5		6	
**Construct 3: satisfaction^d^**					
	**System Usability Scale score per round^e^**				
		**Round 1**			
			Values, n (%)	5 (23)	
			Values, mean (SD)	91.6 (4.23)	
			Margin of error	4.46 or –4.46	
		**Round 2**			
			Values, n (%)	6 (27)	
			Values, mean (SD)	87 (10.23)	
			Margin of error	12.72 or –12.72	
		**Round 3**			
			Values, n (%)	5 (23)	
			Values, mean (SD)	79.5 (16.91)	
			Margin of error	21.02 or –21.02	
		**Round 4**			
			Values, n (%)	2 (9)	
			Values, mean (SD)	78.8 (18.75)	
			Margin of error	N/A^f^	
		**Round 5**			
			Values, n (%)	4 (18)	
			Values, mean (SD)	78.8 (5.73)	
			Margin of error	9.11 or –9.11	

^a^Quantitative data show the number of participants in each round and the percentage that achieved the construct theme.

^b^Time taken to complete an assessment (time taken to reach a disposition was not measured during round 1, as the “think-aloud” method of data capture was prioritized at this stage); number of system errors where the participant was unable to navigate to the end of the assessment because of a system technical error; backsteps where the participant moved back to the previous question.

^c^DART: Digital Assessment Routing Tool.

^d^System Usability Scale scores by round, group of interest, and across all groups.

^e^Responses were scored on a 5-point Likert scale (1=strongly disagree and 5=strongly agree) and converted to a score of between 0 and 4, with 4 being the most positive usability rating. Converted scores for all participants are multiplied by 2.5 to give a range of possible total values from 0 to 100. We used 90% CI to allow the benchmarking of the overall DART System Usability Scale score with other studies using this value [46].

^f^N/A: not applicable.

**Table 7:** System Usability Scale score per group for construct 3 (satisfaction) of the International Organization for Standardization 9241-210-2019 standard.

System Usability Scale^a^ score per group	Daily internet users (n=19)	Infrequent internet users (n=3)	ESOL^b^ internet users (n=6)	All participants (n=22)
Values, mean (SD)	86.5 (4.48)	70.8 (5.44)	78.1 (4.60)	84.3 (12.73)
Margin of error	1.78 or –1.78	9.17 or –9.17	3.79 or –3.79	4.67 or –3.79

^a^Responses were scored on a 5-point Likert scale (1=strongly disagree and 5=strongly agree) and converted to a score of between 0 and 4, with 4 being the most positive usability rating. Converted scores for all participants are multiplied by 2.5 to give a range of possible total values from 0 to 100. We used 90% CI to allow the benchmarking of the overall Digital Assessment Routing Tool System Usability Scale score with other studies using this value [46].

^b^ESOL: English for speakers of other languages.

### Effectiveness

**Figure 10:**
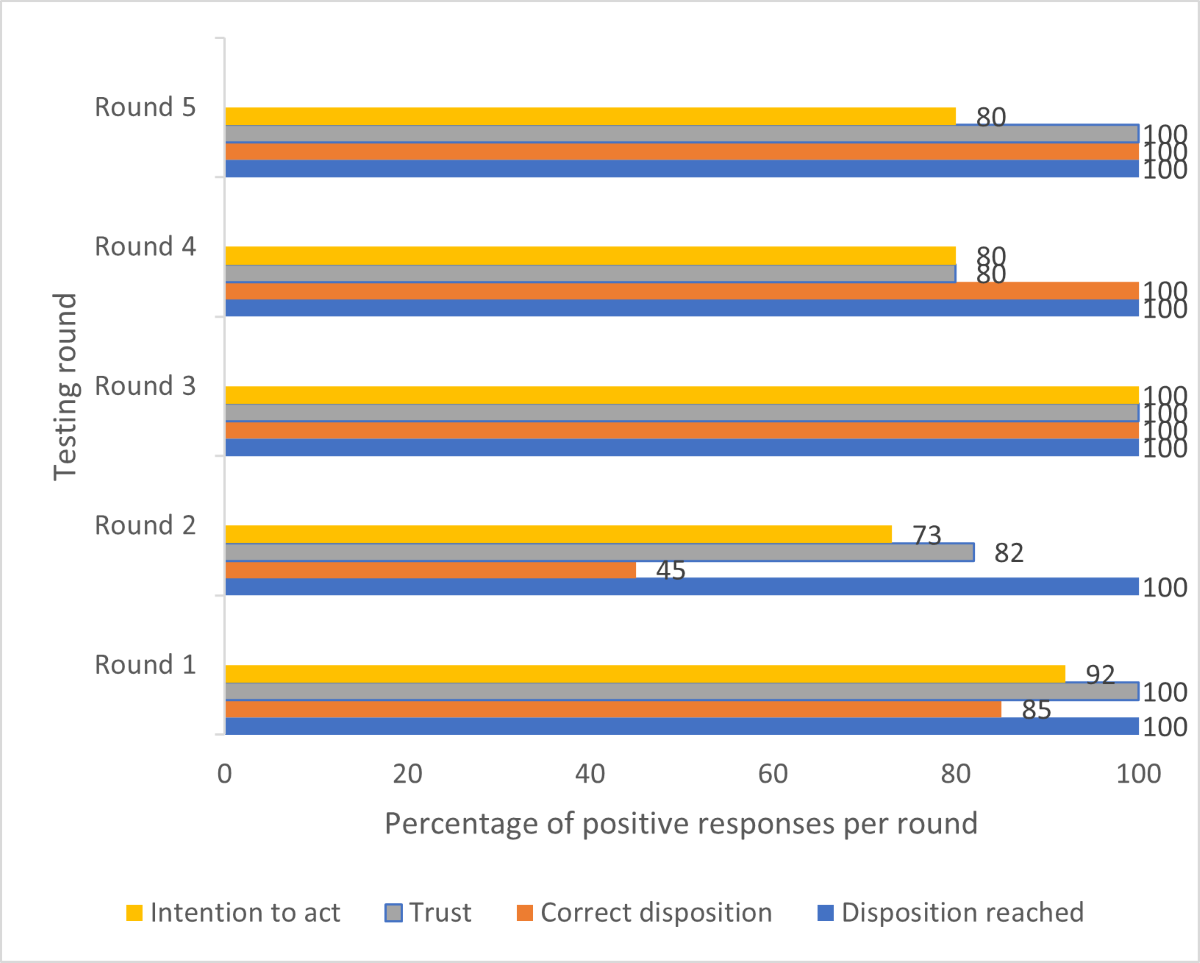
Combined measures of effectiveness by testing round. Results are displayed as the percentage of total assessments to allow comparison, as there were different numbers of participants and assessments in each round. The percentage of assessments in each round resulted in a positive response to the following queries: (1) whether a disposition was achieved, (2) whether it was a clinically correct disposition, (3) whether the participant would trust the disposition, and (4) whether the participant would act on the disposition.

All assessments resulted in a recommendation. Other measures of effectiveness improved over the DART iterations, culminating in a high degree of efficiency being reached (Figure 10).

Of the 50 assessments, 8 (16%) resulted in an incorrect recommendation being given, equating to a grade 1 usability issue. Qualitative data revealed that the selection of the incorrect body site at the start of the assessment was responsible for one of these errors. A total of 7 inappropriate clinical escalations were triggered by 2 specific screening questions for systemic inflammatory disease (SID) and central nervous system conditions. Both were reviewed against the evidence base, rewritten, and incorporated into iteration 3. Subsequent testing rounds, including inviting the participants who revealed this problem to retest, confirmed that this usability issue was solved. Participants said their trust and willingness to act would increase if all their symptoms are considered, and this could be achieved by adding a text box on the body site page where they could enter information about problems in other body areas. A related theme was participants wanting to personalize their assessment by adding additional information, and DART iteration 3 included the addition of a free text box at the end of each page. This improved both trust and intention to act, with all participants during testing round 5 arriving at a correct recommendation that they would trust, with just one assessment where the participant said they would not act on the recommendation related to their previous experience of their MSD resolving spontaneously. A small number of participants felt that the lack of personalization of the DART recommendation page made them less likely to act on the advice. This was a result of a test version being used for the study, containing a simple generic recommendation rather than the detailed advice and next actions that would be found on a production version. However, the importance of this feedback was noted and will guide the final DART version to be deployed.

### Efficiency

Quantitative indicators of efficiency remained high throughout testing, reinforced by qualitative data.

**Figure 11:**
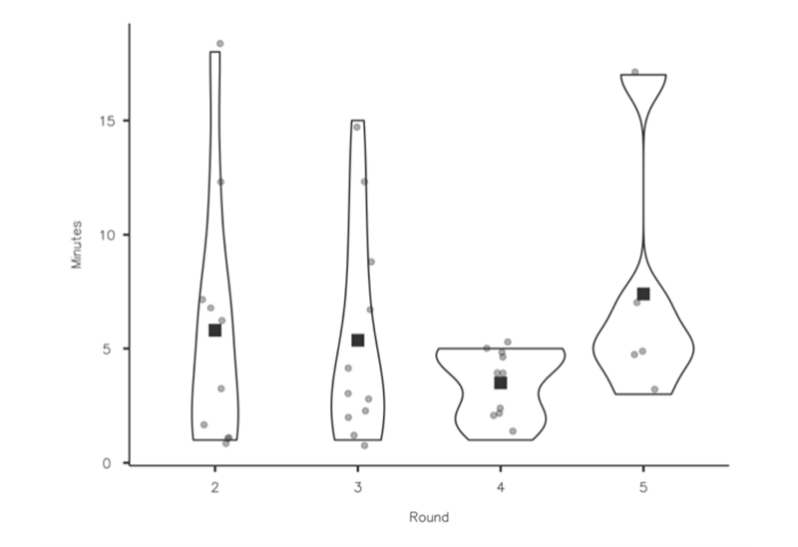
Efficiency (time to complete an assessment). Time taken for participants to complete assessments. A total of 16 participants completed 34 assessments in total across rounds 2 to 5. Time was not recorded in round 1, as participants were encouraged to use the “think-aloud” technique.

Round 5 had a larger number of ESOL participants, and it was noted the mean time for this group was slightly longer (6 minutes). The longest time (18 minutes) taken to complete an assessment was by an ESOL participant (Figure 11). All participants without exception said that the time taken to complete an assessment was acceptable and that the format of the questions was clear and supported their ability to make decisions easily.

System errors were defined as DART technical errors, such as presenting the user with an error message or the system timing out. No system errors were encountered during any testing rounds.

**Figure 12:**
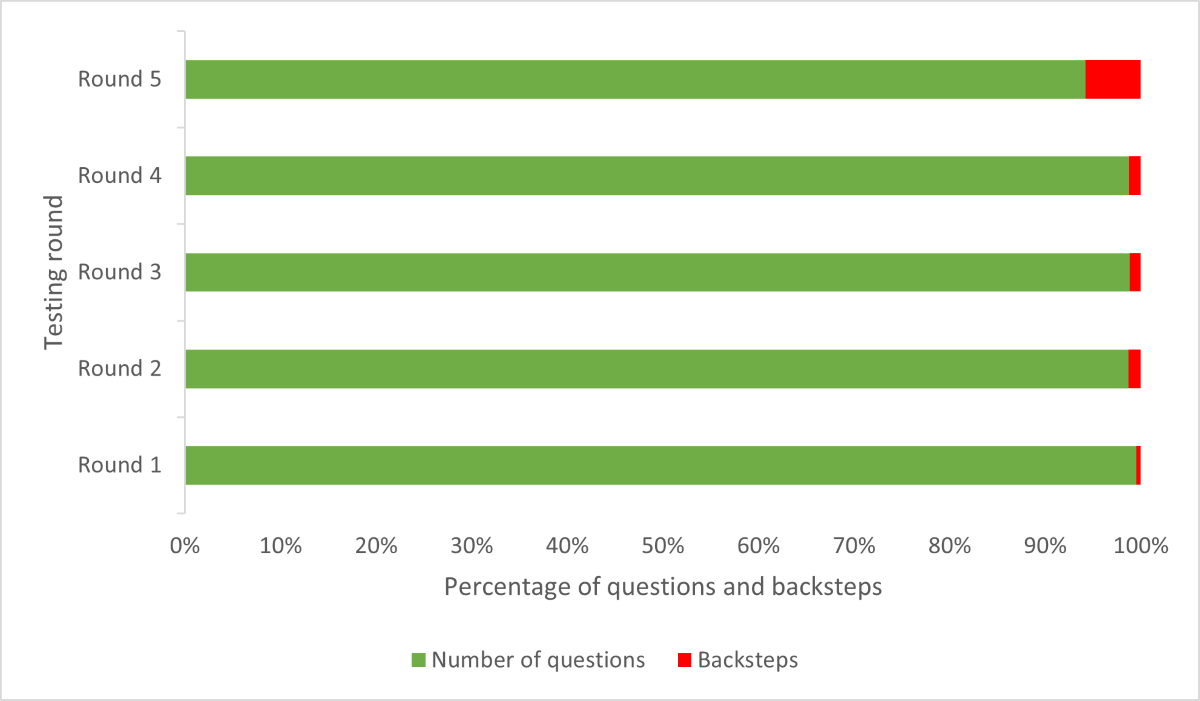
Measures of efficiency (backsteps) by testing round. Number of times a participant moved back a step in the question set to review their previous question and response, with backsteps shown as a percentage of the total number of questions in the assessment.

The number of times a participant moved back in the pathway to review the previous question remained consistent across the first 4 testing rounds, even with the introduction of participants who used the internet less frequently and ESOL users. Backsteps increased to 6 in round 5 and were linked to one of the ESOL participants, who had the lowest recorded eHEALS score (Figure 12). He told us some of the questions were more challenging to answer and required him to reread them.

### Satisfaction

**Figure 13:**
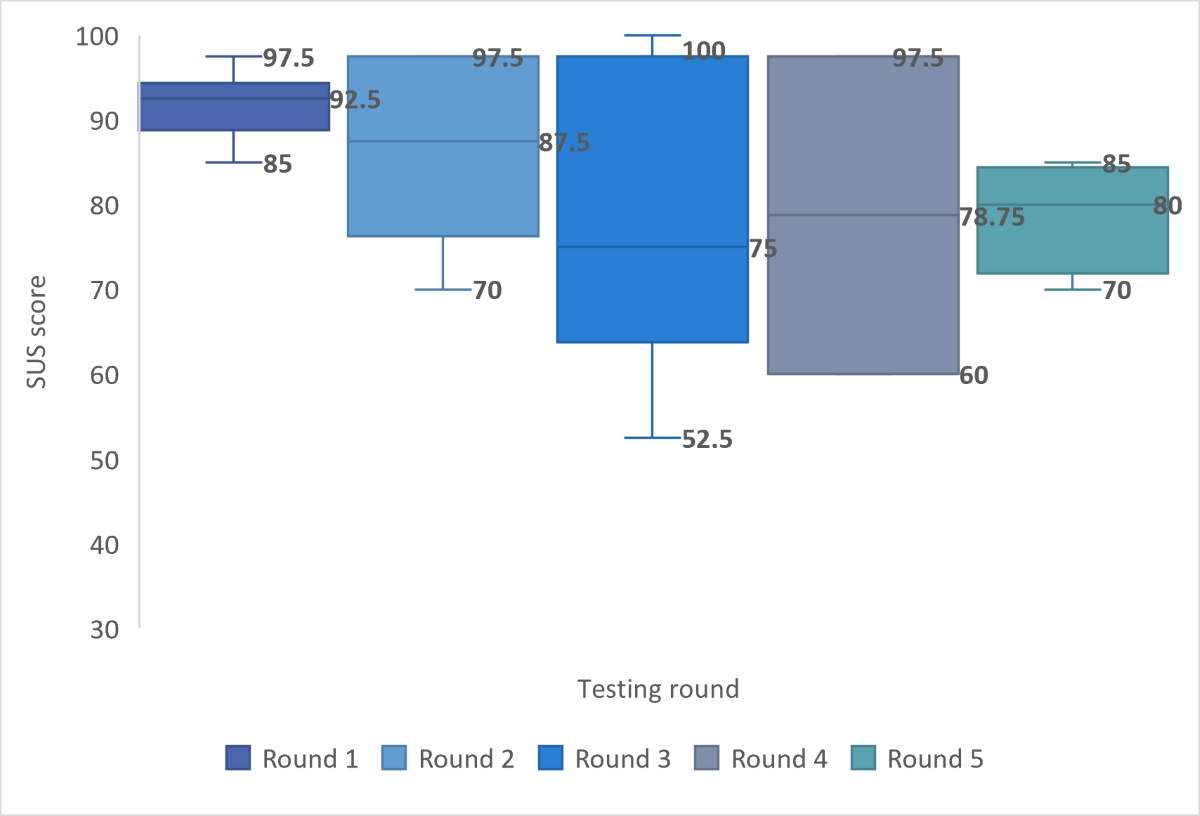
Measure of satisfaction by testing round. SUS: System Usability Scale.

Satisfaction was measured quantitively across all groups using the SUS, with qualitative data providing deeper insights into specific question responses. Although high levels of system satisfaction were prevalent throughout testing, cumulative satisfaction scores reduced with each round as infrequent internet users and ESOL participants were recruited (Figure 13). However, the final mean SUS score of 84.3 (SD 12.73; 90% CL 4.67) across all groups achieved the predefined study end point of a score of ≥80, representing a “good” or better system and associated with an increase in the probability that users would recommend DART to a friend [37].

Differences in mean satisfaction scores were present between groups, with daily internet users scoring highest (19/22, 86%; mean 86.5, SD 4.48; 90% CL 1.78 or –1.78), and nonnative English speakers (6/22, 27%; mean 78.1, SD 4.60; 90% CL 3.79 or –3.79) and infrequent internet users scoring the lowest (3/22, 14%; mean 70.8, SD 5.44; 90% CL 9.17 or –9.17), although the CIs overlap.

Although care should be taken in examining individual SUS item responses as external validity only exists on aggregated scores [51], analysis of the highest scoring questions did reveal some useful insights. All participant groups scored highest when saying that they would not need to learn many things before they could use DART. Both daily and infrequent internet users agreed they would not need technical support to use DART, with ESOL participants agreeing to a slightly lesser extent.

All groups did not feel they would use DART frequently or that the functions in the system were well integrated. This was not an unexpected finding as DART is intended for single-time use to determine the correct level of intervention for the user’s MSD and, therefore, would not be used frequently. In contrast to most other mHealth systems, DART is not designed to provide an MSD intervention, with no requirement for the user to navigate to additional features within the system.

Satisfaction adjectives were associated with each participant’s individual total score to aid in explaining the results to non–human factor professionals [52], with 91% (20/22) of participant scores equating DART as a “good,” “excellent,” or “best imaginable system.” The remaining 2 (8%) participants rated DART as “fair,” with none rating it as “poor” or “worst imaginable.”

Using the normalizing process described by Sauro [46], DART ranks within the 96th to 100th percentile (SUS score 84.1-100) of systems tested using the SUS, with an associated adjective rating of “Excellent” [53]. Benchmarking of the DART SUS score against the mean score of 67 (SD 13.4) from 174 studies assessing the usability of public-facing websites utilizing the SUS revealed that DART was among the highest scoring systems assessed in this way [46].

### Accessibility

Accessibility has been central to the design of the DART user interface, and the Appian platform on which DART is constructed includes features supporting accessibility for a wide range of users, including those with disabilities who use assistive technologies such as screen readers.

DART’s simplicity of use was recognized in the qualitative data by all participants from all groups, who felt that it was sufficiently simple to use and liked the fact that all assessments resulted in a disposition being given. This supports the theory of a low barrier to entry for DART, provided users have internet access:

It is so simple, its one most simple of the things, websites, I've engaged with.

DART016

It was very quick. And I quite like that it has one thing for one page, which is a very short question, it gives you a few options, and then you answer so you don't have to go through long text questions, one after the other. So, it just takes you very quickly step by step. And it's quite, I don't know, for me, it was super easy and clear to answer questions.

DART017

We asked participants whether they could think of anyone who may not be able to use DART:

I wonder how somebody like my mom's age would cope with it and I actually thought there wouldn’t be too many who wouldn’t.

One of the ESOL participants tested DART with the help of her daughter, which she told us was her normal practice when she needed to use the internet and common practice within her community:

No it’s easy to do. My daughter is helping me. Little bit, I understand most of the things, but little bit some questions, what can I say? So, my daughter guides me and help me.

DART012

When asked how other ESOL users would use DART, she said the following:

I think they need somebody's help, their partner or their children, somebody, or some of their friends, some can help them and then they can do it.

Overall, participants felt that DART would be easily accessible to people who are familiar with using the internet but that a telephone-delivered alternative would be required for some users. Additional benefits of reducing the time to receiving a diagnosis or treatment and financial savings were also mentioned:

If I had this actual system, I would have saved £150 in cash and probably three months of pain had I been able to access it when I had my problems with my back.

DART013

## Discussion

### Principal Findings

The use of the iterative, convergent mixed methods design proved effective; rich data provided objective measures of system performance together with identification of serious usability problems and solutions by real-world users. The results from this study indicate that through a series of iterations, DART usability reached a sufficiently high standard to proceed to further safety and effectiveness trials.

#### Theme 1: Factors Leading to an Incorrect Recommendation Being Given

Selection of the appropriate body site is crucial to driving matched clinical algorithms within DART, and failure to do so accounted for 8 incorrect recommendations. In addition, participant confidence in DART being able to recognize their body site selection was considered important to most participants, being related to their wider trust in the system and associated intention to act on the recommendation they received. As a result, the body site diagram was refined across all iterations.

A specific DART question designed to screen for SID triggered false-positive recommendations for participants describing mechanical pain. Correct identification of SID can be problematic for primary care clinicians because of varied symptom presentation and overlap with more common osteoarthritic joint conditions [53-55]. A study using patient-entered responses showed that osteoarthritis was diagnosed in 38% of SID cases [56], a result that is clinically significant, given the interdependency of early recognition of SID, minimizing a poor patient outcome [57,58]. During DART testing, these participants prioritized the presence of pain characteristics of osteoarthritis over the hot and swollen multiple joint symptoms presented in SID. This problem was addressed by a detailed review of the literature on the differential diagnosis of SID, rewriting, and inclusion of new questions within all algorithms with associated linked age logic. Subsequent testing rounds, including participants who revealed this problem, confirmed success in negating this usability issue. The implications of creating false positives are often underestimated, with most symptom checker development taking a conservative approach, resulting in systems typically being more risk averse than health care practitioners [59-61]. However, the failure to provide an accurate routing decision can affect user trust and system adoption [62], as well as the creation of unnecessary referrals to urgent or emergency services.

#### Theme 2: Impact of Assessment Personalization on Participant Trust and Intention to Act on the Given Recommendation

Personalization of assessments was perceived by some participants as a key advantage of a patient-clinician triage interaction over an mHealth system. It has been shown that lack of personalization can affect trust and intention to act, with implications for system adoption [63]. Our participants wanted to “tell their story,” not feeling involved in the assessment process unless they could provide information personal to them, including entering details of secondary body site areas. It has been estimated that 8% of patients with MSD presenting to a primary care physician have a problem in >1 body site [64]; thus, an additional comment box was added to the body site diagram page, inviting users to enter other problem areas, something our participants said would address this problem.

In all but 1 assessment, participants who said they would trust DART also said that they would act on the recommendation they were given. A direct correlate of eHealth user trust is information quality, defined by knowledgeable, impartial, and expert sources. These are important factors that lead users to believe the system is acting in their best interest, as they feel a clinician would do [65].

Trust factors have a significant direct effect on user intention to act [66], a key requirement for successful DART introduction into an MSD digital pathway and system adoption. However, we found other factors may be at play, such as previous experience in MSD management. One of the participants told us that they would trust the DART recommendation for physiotherapy but would have waited to see whether their problem improved without treatment, as it had before. It has been recognized that when users of eHealth make decisions on system trust and intention to act, especially those with high eHealth literacy, they will often corroborate information using other web-based content to “triangulate” advice, particularly if the primary source is not familiar to them [63,66]. Interestingly, it has been shown that eHealth users in the United Kingdom with access to free NHS health care are less likely to use health corroboration than users in other countries with private health systems [66]. The NHS website is considered a trusted source of information for many citizens in the United Kingdom, and deploying DART within an NHS pathway may enhance a user’s trust in the given recommendations.

Qualitative data revealed a usability problem that was not considered during development—that serious condition-screening questions on DART had the potential to cause user anxiety in some individuals, who otherwise would not have considered the potential for their problem to be serious. On the basis of this feedback, we placed these less frequently occurring conditions in the context of their incidence to allay unnecessary user anxiety. This is an important consideration for developers of mHealth triage systems, as although the creation of false positives is well recognized and largely accepted as a prudent conservative approach to medical risk management [62], it is also suggested that a significant proportion of potential users would reject using a symptom checker for fear of receiving a wrong diagnosis or an assessment that could cause them anxiety [60].

#### Theme 3: Impact of User Interpretation When Answering Specific DART Questions

A small number of questions provoked some unexpected participant responses attributable to individual interpretation, likely influenced by their personal experiences. This theme was not identified during previous validation work completed by the panel of expert clinicians. It was only possible to reveal and understand this important usability factor by using a convergent mixed methods design with real-world users, reinforcing the advantage of this methodology over the common practice of using vignettes constructed or delivered by clinicians. An expert clinician recognizes conditions by virtue of pattern recognition, bypassing the conscious, effortful cognitive requirements demanded of a nonclinician user to interpret questions and make decisions on how to respond [67]. Moreover, clinicians are highly educated and not representative of a real-world system user population, including people with eHealth literacy challenges. This study concluded that diversity of user personal experiences can influence how real-world users respond to questions presented by an mHealth system and, ultimately, the recommendation they receive, thus presenting a challenge to developers. For this reason, it is suggested that clinical testing of mHealth systems using vignettes is best used as a precursor to real-world usability testing comprising a representative sample of potential system users.

We found no relationship between age and eHealth literacy, with older participants equally able to arrive at a recommendation as the younger participants. Although this finding should be treated with caution because of the small number of older participants, it could suggest that the perceived ability to seek and use health information is more related to the frequency of internet use rather than age and that differences in eHealth literacy are less likely to be between user group demographics but rather socioeconomic variables between individuals within them [68]. A recent report showed continued growth of internet use in the United Kingdom, with a 6% increase in households with internet access between 2018 and 2020. In the same period, the increase in the number of households with a single adult aged >65 years who accessed the internet within a 3-month period rose from 59% to 80% [39], challenging perceptions about potential mHealth user demographics. An ESOL participant who had assistance from her daughter told us she often sought help from family or neighbors to use the internet, that this was common practice within her community, and that DART could be used effectively in this way. Web-based “surrogate seeking” is now a widespread practice, with significant numbers of internet health information seekers accessing advice on behalf of someone else [69]. However, some studies still link the use of web-based symptom checkers to younger and more highly educated populations [60] and self-referrals for the assessment of musculoskeletal conditions generally [70].

### Limitations

Recruitment during the COVID-19 pandemic proved challenging, particularly for people who were not daily users of the internet, as they typically do not engage with social media or advertisements sent via email. All data had to be gathered remotely, affecting the recruitment of people not confident in using web-based video call technology. Although the full recruitment quota was not met for infrequent internet users, this was partially addressed, and the feedback from these participants was particularly valuable in highlighting usability problems. This recruitment challenge could be an indicator of self-selection for DART user adoption related to internet use.

During the DART tests, most participants recalled past conditions that had been resolved or that had been present for some time and had changed since the first onset. At times, this created a problem for participants regarding how to respond to questions; for example, current symptoms versus symptoms they had at the beginning of their problem when they first sought clinical advice. This could be addressed in future studies by only recruiting participants with current problems who had not received medical advice; however, this could potentially exclude participants with chronic MSDs and more complex conditions, thus limiting generalizability.

Although a generic internet system assessment tool, the SUS was chosen as a measure of DART user satisfaction in the absence of a more specific validated mHealth usability measure. As a result, not all the questions were matched to DART in its role as a single-use assessment system with no additional integrated functionality. Other usability assessment tools were considered, including those that measure domains such as loyalty, trust, credibility, and appearance; however, these were designed for the assessment of transactional business systems and included questions inappropriate for DART, such as purchasing and confidence in concluding business [37]. Other tools measured the usability of mHealth systems that support the therapeutic management of conditions over time, with repeated patient use and different integrated functions and features, meaning that the domains assessed were not directly applicable [71].

### Future Work

The purpose of this usability study was to optimize usability before proceeding to a trial evaluating the safety and effectiveness of DART against a usual care comparator. A protocol for an initial pilot study has been published and will explore the key aspects of the trial methodology; assess the procedures; and collect exploratory data to inform the design of a definitive, randomized, crossover, noninferiority trial to assess DART safety [36]. DART is currently deployed in a controlled live clinical environment where we use system data, as well as user and clinician feedback, to further refine the algorithms and system usability. A quality improvement study, where DART is integrated into an existing public health service, is also in the design phase.

### Conclusions

This study suggests the DART mHealth system has the potential to be offered as an alternative to primary care physician–led or physiotherapist-led triage as part of an MSD pathway. Participants found DART easy to use and would trust and act on the routing recommendation they were given. With all significant usability problems addressed, DART can proceed to the next stage of validation—a randomized controlled trial to assess the safety and effectiveness against a usual care comparator. The inclusion of real-world participants revealed important usability problems and solutions that were not identified during the development or expert panel review stages and highlights the importance of a more sophisticated approach to mHealth system usability testing. The iterative, convergent mixed methods design proved to be highly effective for system development and evaluation and could provide a blueprint for other researchers of mHealth systems.

## Appendix

Multimedia Appendix 1 Visual joint display (researcher version).

## Abbreviations

CL: confidence level
DART: Digital Assessment Routing Tool
eHEALS: eHealth Literacy Scale
ESOL: English for speakers of other languages
ISO: International Organization for Standardization
mHealth: mobile health
MSD: musculoskeletal disorder
NHS: National Health Service
SID: systemic inflammatory disease
SUS: System Usability Scale
