# Supplementary material for: Usability Testing of a Digital Assessment Routing Tool for Musculoskeletal Disorders: Iterative, Convergent Mixed Methods Study
Source: J Med Internet Res. 2022 Aug 30;24(8):e38352. doi: 10.2196/38352 (PMC9472040; doi:10.2196/38352)
Supplement: Multimedia Appendix 1 [file jmir_v24i8e38352_app1.docx]

|  |  |  |
| --- | --- | --- |
| **Quantitative variables** | Up to 3 assessments | **Qualitative questions (examples only, use quantitative responses to guide questions)** |
| Task completion (arrival at disposition)  Would you trust the recommendation given?  Would you act on the recommendation given? | YES/ NO  YES/NO  YES/NO  yes/no/unsure  yes/no/unsure  yes/no/unsure  yes/no/unsure  yes/no/unsure  yes/no/unsure | - How easy was it to understand the questions? How could we make them easier to understand? - How easy was it to decide on the answer? How could we make this easier? - Would you trust the advice being given? Would you want to check the advice somewhere else? Where/who? - Did you get the recommendation you were expecting?   Would you act on the recommendation given? If not, why not? |
| Did DART give an arguably correct disposition? | yes/no/unsure  yes/no/unsure  yes/no/unsure | Use information from participant and clinical judgement to determine if correct. If not, explore reasons. |
|  |  |  |
| Time taken to arrive at disposition | Min/secs | How do you feel about the length of time it took to complete the assessment? |

**CONSTRUCT - Effectiveness**
